# Comparison of the Morphology and Developmental Potential of Oocytes Obtained from Prepubertal and Adult Domestic and Wild Cats

**DOI:** 10.3390/ani11010020

**Published:** 2020-12-24

**Authors:** Joanna Kochan, Agnieszka Nowak, Wiesława Młodawska, Sylwia Prochowska, Agnieszka Partyka, Józef Skotnicki, Wojciech Niżański

**Affiliations:** 1 Department of Animal Reproduction, Anatomy and Genomics, University of Agriculture, Mickiewicza 24/28, 30-059 Krakow, Poland; nowak.a.a@gmail.com (A.N.); rzmlodaw@cyf-kr.edu.pl (W.M.); 2 Department of Reproduction and Clinic of Farm Animals, University of Environmental Science, Grundwaldzki Square 49, 50-357 Wroclaw, Poland; sylwia.prochowska@upwr.edu.pl (S.P.); agnieszka.partyka@upwr.edu.pl (A.P.); wojciech.nizanski@upwr.edu.pl (W.N.); 3 Foundation Municipal Park and the Zoological Garden in Cracow, ul. Kasy Oszczędności Miasta Krakowa 14, 30-232 Krakow, Poland; nomail@gmail.com

**Keywords:** felids, wild cats, oocytes, IVF

## Abstract

**Simple Summary:**

This study was conducted with the aim of determining the morphological similarities and developmental potential of oocytes obtained from adult and prepubertal domestic cats (*Felis catus*) and wild cats. The results of our research showed that ovaries obtained from prepubertal felids may be a rich source of good quality oocytes that are competent for in vitro maturation and able to reach blastocyst stage after in vitro fertilization. The results are important in the context of the possibility of using oocytes from prepubertal felids threatened with extinction in conservation programs based on assisted reproductive techniques (ART).

**Abstract:**

The aim of the study was to compare the morphology and developmental potential of oocytes obtained from adult and prepubertal domestic cats (*Felis catus*) and wild cats (*Lynx lynx*, *Leptailurus serval*, *Felis manul*, *Panthera tigris altaica*). The average number of oocytes obtained from an adult domestic cat was 23 ± 11, which was significantly lower than from kittens (43 ± 29). A similar number of oocytes was derived from adult Pallas’s cats (28 ± 8), and serval (30). The lowest number of oocytes was collected from the lynx (5 ± 3). No oocytes were obtained from newborn Amur tiger while in the case of older domestic and Pallas’s cat and lynx kittens (1–3 months) 43, 48 and 41 oocytes were collected, respectively. Significant differences (*p* < 0.001) were observed between the number of oocytes with dark cytoplasm from adult and prepubertal animals of all analyzed species. The diameter of oocytes from adult and prepubertal animals was similar in all species, and was on average 161 ± 4 µm for oocytes with dark cytoplasm and 150 ± 18 µm for oocytes with light cytoplasm. In all species, oocytes with light cytoplasm were significantly smaller (*p* < 0.05) than dark ones, and their population was more diverse. Results of in vitro maturation of the domestic and wild cat′s oocytes obtained from adult and prepubertal females were similar (47–52%). The cleavage rate after in vitro fertilization (IVF) was lower for prepubertal than adult domestic cats (42 vs. 51%; *p* < 0.05%). Moreover, we observed differences in the quantity (28 vs. 39%; *p* < 0.05) and quality of blastocysts and even greater problems with hatching blastocysts from prepubertal kittens (8 vs. 19%; *p* < 0.001). More blastomeres were detected in blastocysts of adult cats. They also demonstrated significantly higher number of inner cell mass (ICM) (*p* < 0.001) and higher number of trophoblast cells (TE) (*p* < 0.05).

## 1. Introduction

Wild felidae are among the most endangered species in the world. Endangered wild cat species conservation programs implemented all over the world are being supported by the latest developments in the assisted reproductive technologies (ART) [1].

Domestic cats (*Felis catus*) are frequently used for research as a model for felid species that are threatened with extinction. Actually, many assisted reproduction methods are tested using the domestic cat model (*Felis catus*) due to their widespread availability of this research material [2,3,4]. Over the last few years, the main focus of research has been to improve in vitro techniques for the intracytoplasmic sperm injection (ICSI) method and for cloning of domestic cats in order to apply these results to wild cats [3,4,5,6,7,8].

Progress in the development of biotechnological methods of assisted reproduction relies on the ability to obtain a sufficient pool of gametes, both oocytes and sperm. Gametes of domestic cats are easy to acquire as a result of the considerable popularity of programs for pet population control that provide a great amount of biological material from thousands of gonadectomies performed in many countries every day.

The availability of gametes from wild felines is very limited. The progress in the studies of semen of wild felines has been achieved mostly as a result of obtaining semen by relatively easy to preform procedures such as electroejaculation or urethral catheterization on the occasion of veterinary treatments [9,10,11,12,13,14]. Unfortunately, obtaining wild cat oocytes is much more difficult. Oocytes may be obtained using laparoscopy after previous hormonal stimulation of ovaries. However, this requires access to a well-equipped operating room and specially trained staff. Laparoscopic aspiration enables acquisition of approx. 10 oocytes from a single cat, which can be used for in vitro fertilization or cloning [15,16]. Oocytes can also be obtained post mortem through ovarian dissection, but these methods of oocyte collection are only used with old or sick females. Ovaries collected post mortem can be stored at 5 °C for 24 h without changes in developmental competence [10,17]. Feline oocytes can be retrieved post mortem using an aspiration procedure involving drawing up the follicular fluid with a syringe and needle or by ovarian scarification involving slitting the cortex in a suitable medium. Ovarian scarification is preferred in cats that have small ovaries. If the oocytes retrieved are immature, they must be matured using in vitro methods. Depending on the condition, age and health of the female, the number of oocytes obtained post mortem ranges from zero to 102 in different wild feline species [17]. When it comes to the domestic cat, the situation is quite the reverse and oocytes can be obtained in large numbers from selected healthy animals of optimal breeding age. The research material obtained from wild felines is so valuable and scarce that selection criteria must be compromised and the ovaries are collected from very young, very old, sick or even dead female individuals irrespective of the cause of death.

As we know, the number of oocytes, their quality and developmental capacity decrease with increasing age [18]. However, the quality and developmental capacity of oocytes obtained from very young kittens have not been studied, even though mortality rates at that age are quite high and thus, it would be possible to retrieve their oocytes for research. Due to the high mortality rate in newborn and very young wild cat kittens kept in zoos, we have the ability to obtain their oocytes and use them for assisted reproduction techniques or to preserve in cell banks.

The developmental ability of oocytes from prepubertal animals has been the object of several studies and successful in vitro embryo production and the birth of live offspring from oocytes of prepubertal animals has been reported [19,20]. Some studies indicate a lower developmental competence [21,22], whereas others describe outcomes similar to adult oocytes [19,23].

Shortage of oocytes from wild felids makes it necessary to test most in vitro embryo production techniques on oocytes obtained from the domestic cat. That is why the efficiency of cryopreservation of wild cat semen was confirmed by conducting reproductive trials using oocytes from the domestic cat [10,11,12]. What is more, in the procedure of somatic cloning, oocytes from the domestic cat can be used as recipient cells. Interspecies cloning proved to be successful as the fusion of African wildcat fibroblast nuclei with domestic cat enucleated oocytes produced African wildcat kittens [2].

The effectiveness of methods applied in in vitro production, such as in vitro fertilization (IVF), ICSI assisted fertilization or somatic cloning, depends largely on the quality of the oocytes, their selection and appropriate in vitro maturation (IVM) conditions. To adapt ART programs for the protection of wild cats and minimize the risk of failure of these procedures, it is necessary to identify similarities and differences between oocytes from wild feline species and the domestic cat, including discrepancies in their morphology. Moreover, an in-depth understanding of the importance of the morphological features is necessary to ensure that an adequate number of oocytes from the most valuable species, maintained through cryopreservation and storage in cell banks, is available in the future for cloning or in IVF programs [24].

The aim of the study was to compare the morphology of oocytes obtained from adult domestic cats (*Felis catus*) and wild felids represented by the northern lynx (*Lynx lynx*), serval (*Leptailurus serval*) and Pallas’s cat (*Felis manul*) at reproductive age, as well as prepubertal domestic cat (*Felis catus*), Pallas’s cat (*Felis manul*), northern lynx (*Lynx lynx*), and Amur tiger (*Panthera tigris altaica*). The study included comparison of the IVM capacity of oocytes retrieved from adult and kittens of domestic cat, Pallas’s cat (*Felis manul*) and lynx (*Lynx lynx*). Additionally, developmental competence of the oocytes after IVF and quality of blastocyst in prepubertal and adult domestic cats (*Felis catus*) were analyzed.

## 2. Materials and Methods

Media used for IVF, such as “Sperm Air^®^” for sperm capacitation and IVC-CULT^®^ for fertilization and embryo culture in vitro are commercial media for human IVF (Gynemed, Lensahn, Germany).

Other chemicals and reagents used in this study were purchased from Sigma-Aldrich (St. Louis, MO, USA).

### 2.1. Source of Ovaries and Oocyte Collection

Ovaries from 20 healthy adult (cycling > 1 year of age) domestic cats were obtained by ovariohysterectomy conducted at local veterinary clinics. Ovaries from prepubertal domestic cats (up to 3 months old; n = 15) were collected post mortem, after euthanasia from veterinary clinics. Ovaries from adult (*Lynx lynx*, *Leptailurus serval*, *Felis manul*) and prepubertal (up to 3 months old) wild cats (*Pantherra tigris altaica*, *Lynx lynx*, *Felis manul*) were collected post mortem in emergency situations at the Zoological gardens in Poland. All adult wild cat females were of reproductive age (4–8 years), fertile, and produced offspring regularly. The causes of death or euthanasia were: femur fracture, complications during sedation, severe diarrhea, acute pneumonia, rejection by the mother and hypothermia, or poisoning. Oocytes were obtained immediately after death or euthanasia.

Ovaries were transported in Dulbecco′s phosphate buffered saline (DPBS) supplemented with 100 μg/mL streptomycin and 100 IU/mL penicillin at 4 °C in a thermal box.

Cumulus-oocyte complexes (COCs) were obtained by scarification in a washing medium (TCM 199 with Earle’s salts, HEPES-buffered and supplemented with 3 mg/mL BSA, and 0.055 mg/mL gentamycin). Oocytes were then assessed using a stereoscopic microscope and divided into four classes based on the quality and quantity of the cumulus cells:

- oocytes tightly surrounded by at least three layers of cumulus cells,
- oocytes partially surrounded by cumulus cells,
- oocytes surrounded only by the corona radiata,
- degenerated oocytes with morphological defects, with damaged zona pellucida, distorted shape or fragmented cytoplasm.

**Figure 1:**
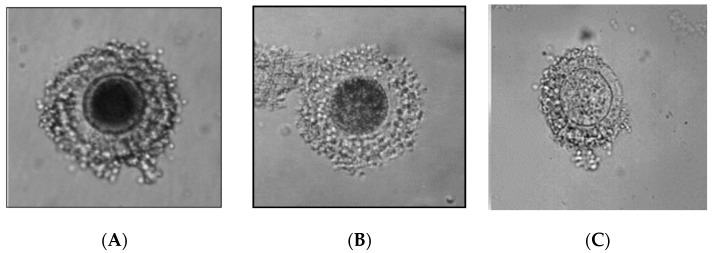
Oocyte classification based on the color of the cytoplasm; (**A**) oocyte with very dark cytoplasm (**B**) oocyte with dark, mosaic cytoplasm, (**C**) oocyte with pale cytoplasm.

The oocytes were also classified according to the color of their cytoplasm (Figure 1):

A- oocytes with very dark cytoplasm,
B- oocytes with dark, mosaic cytoplasm,
C- oocytes with pale cytoplasm,

The numbers of oocytes with dark (A,B) and pale cytoplasm (C) were counted and the oocyte diameter was measured.

### 2.2. In Vitro Maturation of Oocytes (IVM)

Only oocytes with dark cytoplasm (grade A,B), surrounded by compact layers of cumulus cells (grade I,II), were selected for IVM. Selected COCs were placed in 4-well dishes 20–40 COCs per 400 µL TCM 199 with Earle’s salts, supplemented with 0.02 IU FSH/mL and 0.02 IU LH/mL 0.25 mg/mL sodium pyruvate, 0.6 mg/mL sodium lactate, and 0.15 mg/mL L-glutamine, and cultured for 24 h at 38.5 °C and 5% CO_2_ in air. About 20–40 COCs were cultured per well except for the lynx, from which only 5 oocytes were obtained. After IVM, oocytes of wild felids were denuded of cumulus cells by mechanical pipetting in hyaluronidase (80 IU/mL) for 2 min, and only those with a visible first polar body were cryopreserved and stored in a cell bank. Some of the domestic cat oocytes were completely denuded of granulosa cells in order to visualize the polar body and determine the maturation rate. The remaining oocytes were partially denuded and subjected to in vitro fertilization.

### 2.3. In Vitro Fertilization (IVF) and Embryo Culture

Thawed spermatozoa of domestic cats, isolated from cauda epididymis and frozen according to the procedure described by Niżański et al. [25] were used. Before IVF, semen was thawed at 37 °C for 30 s. After that, the semen was centrifuged with “Sperm Air” medium (Gynemed, Lensahn, Germany) and then incubated at 38.5 °C for 30 min to capacitate the spermatozoa. Oocytes were inseminated with 5 × 10^5^ motile spermatozoa/mL in 400 μL IVC-CULT (Gynemed, Lensahn, Germany) medium at 37 °C in 5% CO_2_ in air.

At 16 h after IVF, presumptive zygotes were transferred to 20 µL droplets (5 embryos/drop) of pre-equilibrated CULT medium (Gynemed, Lensahn, Germany) under mineral oil and cultured up to 8 days. Every second day, half the volume of culture medium was exchanged for fresh medium.

### 2.4. Differential Cell Staining and Counting of the Nuclei

Blastocysts on day 7 after IVF were incubated for 15 s in PBS containing 0.5% Triton-X and 100 µg/mL propidium iodide to stain trophoblast cells (TE) in 37 °C. Blastocysts were then immediately transferred into 100% ethanol with 15 mg/mL Hoechst 33,342 and incubated for at least 40 min in 4 °C to stain inner cell mass (ICM). Cells were mounted onto a glass microscope sidle with a drop of glycerol, flattened with a coverslip, and evaluated using epifluorescence inverted microscope (Nicon Eclipse 200) with UV excitation of 345 and 535 nm resulting in bisbenzimide-stained nuclei fluorescing blue (ICM), whereas propidium iodide-stained cells fluorescing red (TE). The ICM ratio was counted as the proportion of the total cell number of blastomeres (TCN).

### 2.5. Statistical Analysis

Non-parametric data, such as differences in the mean percentage values between groups, were assessed by the chi-squared test. Parametric data, such as oocyte dimension and number of blastomeres in blastocysts, were expressed as means, SD and were compared by two-way ANOVA. Significant statistical difference was noted at *p* < 0.001.

## 3. Results

Table 1 presents data on the number and quality of oocytes collected from the adult and prepubertal domestic and wild cats. The average number of oocytes obtained from an adult domestic cat was 23 ± 11, which was significantly lower than from kittens (43 ± 29). A similar number of oocytes was derived from adult Pallas’s cats (28 ± 8), and serval (30). The lowest number of oocytes was collected from the lynx. Although all adult females were of comparable age (4–8 years) and the oocytes were collected in the same season, the average number of oocytes from lynx was only 5 ± 3. No oocytes were obtained from newborn domestic cats or from Amur tiger cubs on the day of birth, while in the case of older domestic and Pallas’s cat and lynx kittens (1–3 months) 43, 48 and 41 oocytes were collected, respectively.

**Figure 2:**
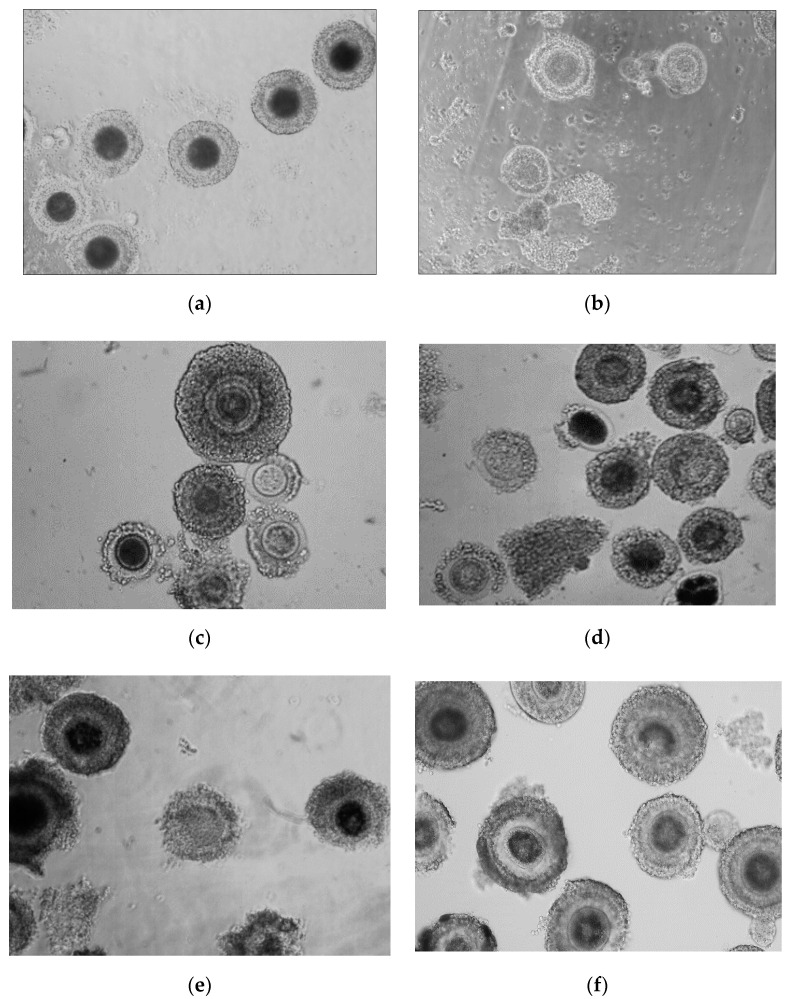
Oocytes of wild felids; (**a**) oocytes with dark cytoplasm from adult serval, (**b**) oocytes with light cytoplasm from adult serval, (**c**) oocytes from adult Pallas’s cats, (**d**) oocytes from prepubertal Pallas’s cats, (**e**) oocytes from adult lynx, (**f**) oocytes from prepubertal lynx.

Significant differences (*p* < 0.001) were observed between the number of oocytes with dark cytoplasm from adult and prepubertal animals of all analyzed species (Figure 2). In adult cats (domestic cat, serval and Pallas’s cats) there were no significant differences between species in the percentage of oocytes with dark cytoplasm (73–80%).

The diameter of oocytes with dark cytoplasm from adult and prepubertal animals was similar in all species, and was on average 161 ± 4 μm.

In all species oocytes with light cytoplasm were significantly smaller (*p* < 0.05) than dark ones, and their population was more diverse with an average diameter of 150 ± 18 µm, but similar for all individuals from a species.

Table 2 presents the results of IVM of oocytes obtained from adult and kittens of domestic cats, Pallas’s cats and lynx. Similar results (*p* > 0.05) were obtained from the adult and prepubertal females, regardless of species (47–52%).

**Figure 3:**
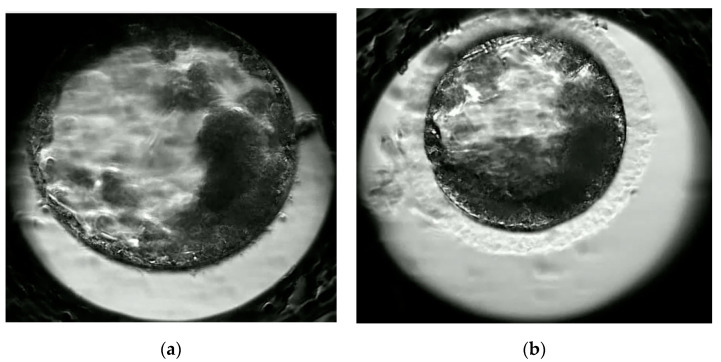
Blastocysts of adult (**a**) and prepubertal (**b**) domestic cat at 165 h post in vitro fertilization (IVF).

Table 3 presents data on the developmental competencies of oocytes after in vitro fertilization. The cleavage rate was lower for prepubertal than adult cats (42 vs. 51%; *p* < 0.05%). Moreover, we observed differences in the quantity (28 vs. 39%; *p* < 0.05) and quality of blastocysts (Figure 3) and even greater problems with hatching blastocysts from prepubertal kittens (8 vs. 19%; *p* < 0.001). More blastomeres were detected in blastocysts from adult cats (Table 4). They also demonstrated significantly higher number of inner cell mass (ICM) (*p* < 0.001) and higher number of trofoblast cells (TE) (*p* < 0.05).

## 4. Discussion

Implementation of reproductive biotechnologies to breed wild felids is extremely difficult. It is seriously hindered by limited knowledge on the physiology of reproduction of particular species of wild cats, poor gamete quality, and a lack of optimized ART treatments that have been developed mostly by using the domestic cat as a model animal [9,24]. Wild felines that are kept in zoos individually often do not produce offspring. Establishing gamete banks covering material from wild cat individuals would reduce the necessity of transporting animals over long distances in order to mate, as well as increase the available gene pool and improve the genetic diversity of a given wild feline species. Unfortunately, despite significant research into methods of in vitro embryo production using domestic cats as a model for nondomestic felines, they are still far from being optimized and many discrepancies are reported in the literature concerning the effectiveness of these procedures [26,27,28,29]. The lack of knowledge concerning the similarities and differences between oocytes of wild felidae species and those of the domestic cat limit the development of ART for the genetic management of domestic and endangered, nondomestic cat populations.

In this study, we obtained an average of 23 oocytes from adult domestic cats, 30 from serval, 28 from Pallas’s cats and 5 from lynx. The available bibliographic resources fail to provide any information on the characteristics of oocytes collected from the lynx and Pallas’s cat. Johnson et al. [17] reported that the number of oocytes decreases in older females. In our study, the age of females ranged between 4 and 8 years. In some cat species, such as the lion (*Panthera lion*), puma (*Felis concolor*), jaguar (*Panthera onca*), and golden cat (*Felis temmincki*) obtaining oocytes from individuals over 14 years old is impossible [17] and is associated with aging and gradual suppression of the sexual function [18]. In other females of comparable age, like the tiger (*Panthera tigris*), leopard (*Panthera pardus*), cheetah (*Acinonyx jubatus*) and clouded leopard (*Neofelis rufus*), oocytes were obtained only from individuals in very good health, but the number of oocytes retrieved was lower than from younger females [17].

There are no references on the collection of oocytes from very young cats. Due to the high mortality rate in newborn and very young wild cat kittens, both living in the wild and kept in zoos, we attempted to determine whether collection of oocytes from young individuals is possible and to compare their quality with oocytes derived from mature females.

In the analyzed species, except for the tiger, the number of oocytes collected from young individuals was significantly higher (*p* < 0.001) than from adults. In the case of the tiger, no oocytes were collected from a newborn cub, while oocytes retrieved from other young cats were morphologically normal and were subjected to IVM.

Previous experiments on cat oocytes revealed that domestic cats develop two types of oocytes: those with dark cytoplasm which are capable of fertilization under in vitro conditions, and those with pale cytoplasm which are characterized by poor fertilization rates. The dark color of the cytoplasm results from the presence of a large quantity of lipids. Significant differences in the number of oocytes with dark cytoplasm were reported for adult and immature cats. Despite collecting a large number of oocytes in total, the presence of a significant number of pale oocytes reduced the quantity of oocytes available for in vitro procedures by about 25% in adults and by about 50% in prepubertal animals. Moreover, only up to 50–60% of domestic cat oocytes reach maturity, which further reduces the number of oocytes displaying developmental capacity [16]. According to the literature, germinal vesicle oocytes collected from prepubertal animals reach the metaphase II stage after IVM at rates similar to oocytes from adult females [22]. Additionally, in our studies, no significant differences were found between the efficiency of IVM of oocytes from adult and prepubertal cats, based on the presence of the first polar body. Polar body extrusion indicates the nuclear maturation of oocyte. However, maturation of the cytoplasm is equally important, as it determines the proper activation of the oocyte, the polyspermy block, fertilization and preimplantation embryo development [30]. In our study, despite oocytes from prepubertal cats being able to reach the metaphase II stage, we noted a decrease in cleavage rate and a significant reduction in their competence to develop to the blastocyst stage and to hatch, which may have been related to cytoplasmic maturity disorders. Similar observations regarding reduced developmental competence of oocytes from very young sheep were noted by Leoni et al. [22] and Ptak et al. [20]. Moreover, according to Leoni et al. [22], prepubertal oocytes reached the MII stage 1 hr later than adult oocytes, and showed higher rates of spontaneous parthenogenetic activation (17.38% vs. 2.08% in adults) and polyspermy (14.30 vs. 2.21%). The frequent occurrence of polyspermy in oocytes from prepubertal females reported by others [31,32] was also noted, possibly as a consequence of the defective dispersion of cortical granules around the cortex. In our experiment, we performed differential staining of blastocysts on day 7 after IVF and observed much fewer cells in blastocysts from prepubertal cats. This may be related to the development delay discussed above.

Despite the problems with in vitro development of embryos from juveniles, it is possible to obtain offspring after embryo transfer to an adult female, as has been proven in cattle and sheep [19,20].

Our experiment confirms that oocytes collected from very young cats are able to reach the blastocyst stage in vitro. Based on the cattle and sheep studies [19,20], we might assume that blastocysts obtained from prepubertal cat oocytes could generate live offspring. This hypothesis should be the subject of further research and a new direction in the use of assisted reproductive techniques in the conservation of wild felines. Due to the critically low population of wild felids, it is important to look for new possibilities to collect and use as much genetic material as possible e.g., in the form of oocytes from prepubertal females. Genetic banks in various institutions already preserve oocytes and semen obtained from diverse cat species, and so should also consider securing oocytes from prepubertal females. Moreover, the creation of a database, to enable exchange of the material between various research centers and thus improve in vitro embryo production techniques, is highly recommended.

## 5. Conclusions

Ovaries obtained from prepubertal, young felids may be a rich source of good quality oocytes that are competent for in vitro maturation and able to reach blastocyst stage after in vitro fertilization.

The results are important in the context of the possibility of using oocytes from prepubertal felids threatened with extinction in conservation programs based on assisted reproductive techniques (ART).

## Figures and Tables

**Table 1:** Characteristics of oocytes obtained from the domestic and wild cats of different age.

Morphology of Oocytes	Adult				Prepubertal			
	Domestic Cat n = 20	Pallas’s cat n = 2	Lynx n = 2	Serval n = 1	Domestic Cat n = 15	Pallas’s Cat n = 1	Lynx n = 1	Tiger n = 1
No. of oocytes/female (mean ± SD)	23 ± 11 ^a^	28 ± 8 ^a^	5 ± 3 ^c^	30 ^a^	43± 29 ^b^	48 ^b^	41 ^b^	0
Oocytes with darkcytoplasm (grade A,B) (%)	77% ^a^	74% ^a^	80% ^a^	73% ^a^	48% ^b^	54% ^b^	53% ^b^	0
Oocytes with light cytoplasm (grade C) (%)	23% ^a^	26% ^a^	20% ^a^	27% ^a^	52% ^c^	46% ^c^	47% ^c^	0
Diameter (µm) of oocytes with dark cytoplasm (mean ± SD)	161 ± 5 ^a^	162 ± 3 ^a^	162 ± 2 ^a^	160 ± 5 ^a^	161 ± 7 ^a^	160 ± 4 ^a^	162 ± 5 ^a^	-
Diameter (µm of oocytes with light cytoplasm (mean ± SD)	152 ± 11 ^a^	154 ± 9 ^a^	152 ± 5 ^a^	153 ± 13 ^a^	153 ± 15 ^a^	151 ± 8 ^a^	152 ± 10 ^a^	-

^a,b^ Within a row, means without a common superscript differ (*p* <0.05). ^a,c b,c^ Within a row, means without a common superscript differ (*p* < 0.001).

**Table 2:** Results of in vitro maturation of oocytes obtained from adult and prepubertal felids.

In Vitro Maturation of Oocytes	Adult			Prepubertal		
	Domestic Cat	Pallas’s Cat	Lynx	Domestic Cat	Pallas’s Cat	Lynx
Total number of oocytes	120	56	10	108	48	37
Oocytes selected for IVM	72 (60%)	23 (64%)	6 (60%)	49 (45%)	25 (52%)	19 (51%)
Oocytes in met II after IVM	38 (52%)	11 (52%)	3 (50%)	24 (49%)	13 (52%)	9 (47%)

No statistical differences were detected within the row for in vitro maturation (IVM) (*p* > 0.05).

**Table 3:** Results of in vitro fertilization (IVF) of oocytes obtained from prepubertal and adult domestic cats.

Group of Females	No. of Fertilized Oocytes n	No of Cleaved Embryos n (%)	No of Blastocyst n (%)	No of Hatching Blastocyst n (%)
Adult	203	104 (51) ^b^	41(39) ^b^	8 (19) ^d^
Prepubertal	218	92 (42) ^a^	26 (28) ^a^	2 (8) ^c^

^a,b^ values with different superscripts within the same column differ significantly (*p* < 0.05). ^c,d^ values with different superscripts within the same column differ significantly (*p* < 0.001).

**Table 4:** Number of blastomeres (mean, SD) in blastocyst from adult and prepubertal domestic cats and its allocation to trophoblast and inner cell mass.

Group of Females	TCN: Total Cell Number	TE: Trophoblast Cells Number	ICM: Inner Cell Mass Number	ICM%
Adult	192 ± 27 ^b^	101 ± 21 ^b^	91 ± 17 ^d^	48
Prepubertal	144 ± 44 ^a^	86± 9 ^a^	58 ± 11 ^c^	41

ICM%-proportion of inner cell mass out of total number of nuclei. ^a,b^ values with different superscripts within the same column differ significantly (*p* < 0.05). ^c,d^ values with different superscripts within the same column differ significantly (*p* < 0.001).

## Data Availability

The data presented in this study are available in this paper. Detailed data on the wild felids used in this study (CITES reports) are available on request from the corresponding author.
